# An Updated Global Perspective of Atrial Fibrillation: Trends, Risk Factors, and Socioeconomic Disparities

**DOI:** 10.1016/j.cjco.2024.12.003

**Published:** 2024-12-15

**Authors:** Sukrit Treewaree, Gregory Y.H. Lip

**Affiliations:** a Liverpool Centre for Cardiovascular Science at University of Liverpool, Liverpool John Moores University and Liverpool Heart and Chest Hospital, Liverpool, United Kingdom; b Department of Medicine, Faculty of Medicine Siriraj Hospital, Mahidol University, Bangkok, Thailand; c Danish Centre for Health Services Research, Department of Clinical Medicine, Aalborg University, Aalborg, Denmark; d Department of Cardiology, Lipidology, and Internal Medicine, Medical University of Bialystok, Bialystok, Poland

Atrial fibrillation (AF) and atrial flutter (AFL) are the most common arrhythmias globally, with far-reaching impacts on quality of life, mortality, and health care costs from stroke, heart failure, and all-cause death, driven by hospitalisations and chronic follow-up.^1^^,^^2^ Previous studies have demonstrated rising global trends in AF prevalence, disability-adjusted life-years (DALY), and deaths, with disparities between different sociodemographic index (SDI) levels; however, some have been limited to specific regions, focused on risk factors, or using dated data.^3^, ^4^, ^5^, ^6^

**Figure 1:**
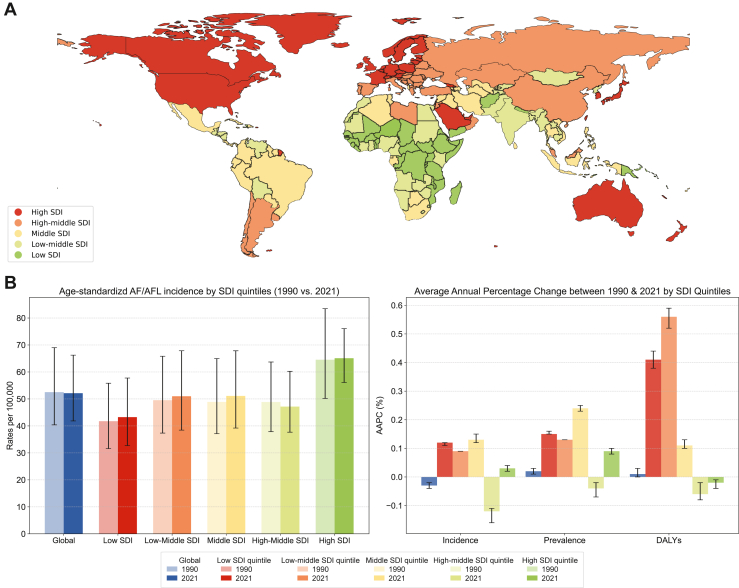
(**A**) The global distribution of sociodemographic index (SDI) quintiles by country. (**B**) Age-standardized AF/AFL incidence rates per 100,000 by SDI quintiles (1990 vs 2021) and average annual percentage change (AAPC) between 1990 and 2021 of age-standardised prevalence, incidence, and disability-adjusted life-years (DALY) rates by SDI quintiles.

Regular updates on the global burden of AF and its complications are essential for guiding public health strategies. Any global view of AF epidemiology should also recognise the ethnic differences in AF-related complications such as stroke and bleeding,^7^^,^^8^ as well as the health inequalities between urban and rural settings within and between countries.^9^ The global distribution of SDI level is shown in Figure 1A.

In this issue of *CJC Open*, Tan et al.^10^ report an analysis of the Global Burden of Disease (GBD) 2021 study that describes the global burden of AF/AFL, focusing on the impact of socioeconomic spectrum, risk factors of AF/AFL, and future projections. The data set used in this analysis includes both primary and secondary data from 204 countries and territories from 1990 to 2021, from a variety of sources, including hospitals, disease registries, censuses, surveys, and health administrative systems, that were used to estimates prevalence, mortality, and DALY across different regions. Future projections of AF/AFL epidemiology were estimated by means of bayesian age-period cohort modelling.^10^

Their analysis found that the global age-standardised prevalence of AF/AFL rose from 1990 to 2021. DALY followed the same trends, as did the age-standardised incidence rate of AF/AFL per 100,000, which was 52.74 (95% confidence interval [CI] 52.72-52.75) and projected to be 54.89 (95% CI 53.55-56.23) in 2035.^10^ The trend of age-standardised incidence of AF/AFL is illustrated in Figure 1B. These updated trends and projections highlight the growing global burden of AF/AFL.

Regarding socioeconomic aspects (based on the SDI, ranging from 0 to 1, which incorporates education, income per capita, and fertility rate), Tan et al.^10^ showed that the highest SDI quintile has the highest age-standardised AF/AFL prevalence,788.35 (95% CI 690.97-910.90) per 100,000, and the lowest SDI quintile has the lowest age-standardised AF/AFL prevalence, 463.23 (95% CI 362.02-602.71) per 100,000. Moreover, a statistically significant positive correlation was observed between SDI and AF/AFL burden, whereby a 0.1 increment in SDI was associated with a 4.94% increase in age-standardised mortality, a 2.56% rise in DALY, and a 2.4% increase in prevalence, with the middle and lower SDI quintiles exhibiting a greater growth in average annual percentage change for prevalence, incidence, DALY, and deaths (Fig. 1B).^10^

This analysis provides a timely update on global burden of AF/AFL, aligning with a previous analysis of GBD 2021 that focuses on risk factors. For example, Cheng et al. demonstrated a similar temporal pattern for age-standardised rate, DALY, and deaths.^4^ Both studies identified high systolic blood pressure and high body mass index (BMI) as major risk factors, with greater effects from high BMI in higher SDI quintiles.^4^^,^^10^ In addition, Tan et al. presented more in-depth insights by showing quantitative analysis of SDI and AF burden, highlighting the relatively increasing burden in lower SDI compared with higher SDI quintiles. The analysis also showed the projection of age-standardised incidence rate and mortality rate in 2035, which could be valuable to policy makers.

Nevertheless, there are some limitations. First, the GBD’s methodology, though comprehensive, might be subject to potential biases due to variations in data quality and availability across different regions. As acknowledged by the authors, the lower prevalence, age-standardised DALY, and death in low SDI quintile vs high SDI quintile might not reflect just the lower AF/AFL burden.^10^ And it might also be confounded by the less robust health data systems and access to health care, leading to potential underreporting. Indeed, one large case-control study of first acute stroke also showed higher pre-stroke prevalence of AF in high-income countries vs lower-income countries, which might be influenced by higher AF screening and access to health care before stroke.^11^ Second, there are limited data from the GBD study regarding the risk factors for AF/AFL and the subtypes of AF/AFL, which prevents a more granular analysis.

Future research directions should focus on addressing gaps in our understanding of AF/AFL burden, particularly in the low to middle socioeconomic spectrum. For example, a prospective cohort study examining the burden of AF/AFL with the integration of granular data on AF/AFL subtypes and region-specific risk factors could be crucial for equitable policymaking and the global mitigation of disease burden disparities.

In addition, the study identifies hypertension and obesity as modifiable drivers of AF/AFL burden, with lower-SDI countries experiencing a steeper rise in AF/AFL burden compared with higher-SDI regions.^10^ Therefore, further investigations into tailored prevention strategies focused on lower-SDI regions such as community-based blood pressure management and obesity-reduction initiatives are warranted. This is well aligned with the current holistic or integrated care approach to AF management, referred to as the Atrial Fibrillation Better Care (ABC) pathway whereby the “C” criterion refers to cardiovascular risk factors and comorbidities, including attention to psychologic morbidity and lifestyle factors. Attention to the latter (ie, healthy lifestyle behaviours) has been shown to be complementary to rhythm control for the reduction of stroke.^12^

The ABC pathway is supported by trial and real-world evidence, where adherence is associated with a reduction in mortality, stroke, bleeding and hospitalisations.^13^, ^14^, ^15^ The ABC pathway is recommended in global guidelines,^16^^,^^17^ but the acronym has recently been modified in the 2023 US guidelines (SOS: stroke, other comorbidities, rate or rhythm control)^18^ and the 2024 European Society of Cardiology guidelines (CARE: comorbidities, avoid stroke, rate or rhythm control, evaluation).^19^ Nevertheless, the various different acronyms (which can informally be referred to as “Easy as ABC” or otherwise “It’s an SOS” or “Handle with CARE”) essentially refer to the main pillars of AF management.^20^

In conclusion, AF and AFL remain significant global health challenges, with an increasing burden reflected in rising prevalence, DALY, and mortality. The study by Tan et al.^10^ provides a comprehensive update on the global impact of AF/AFL, emphasising the socioeconomic disparities and highlighting modifiable risk factors, such as hypertension and obesity. These findings underscore the urgent need for targeted public health interventions and holistic or integrated care management, particularly in lower-SDI regions.
